# Juvenile hormone mimics induce a cellular immune response in *Drosophila melanogaster*

**DOI:** 10.17912/micropub.biology.001797

**Published:** 2025-09-24

**Authors:** Thuan Luu, Johnny R. Ramroop, Min Kyung Lee, Harmeet Kaur, Carolyn W. McGrail, Shubha Govind, Rebecca F. Spokony

**Affiliations:** 1 Biology, City College of New York, CUNY, New York, New York, United States; 2 Natural Sciences, Baruch College, CUNY, New York, New York, United States; 3 Biology and Biochemistry, The Graduate Center, CUNY, New York, New York, United States; 4 Biology, The Graduate Center, CUNY, New York, New York, United States

## Abstract

It is well-known that exposure to juvenile hormone mimics induces a variety of aberrant developmental and physiological effects in insects. One such effect in *Drosophila melanogaster* is the appearance of melanized tumor-like structures in larval stages. To understand the nature of these tumors and identify the constituent cell-types, we examined the effects of two juvenile hormone mimics, methoprene and pyriproxyfen, and found that both mimics induce hematopoietic tumors in flies in a consistent manner. These effects are not observed in flies lacking functional receptors for the juvenile hormone signaling pathway. Using cell-type-specific markers, we found that the juvenile hormone mimic-induced tumors are composed mainly of lamellocytes, a specialized blood cell type that normally differentiates in response to parasitoid wasp infection. Surprisingly however, the larvae without functional juvenile hormone receptors are able to mount a robust encapsulation response when exposed to parasitoid wasps. These results suggest that juvenile hormone mimics require juvenile hormone receptor function to activate cellular immunity and raise new questions about the effect of juvenile hormone mimics on hematopoietic development.

**Figure 1. Juvenile hormone mimics (JHMs) induce melanotic tumors through the induction of specialized hemocytes called lamellocytes:**
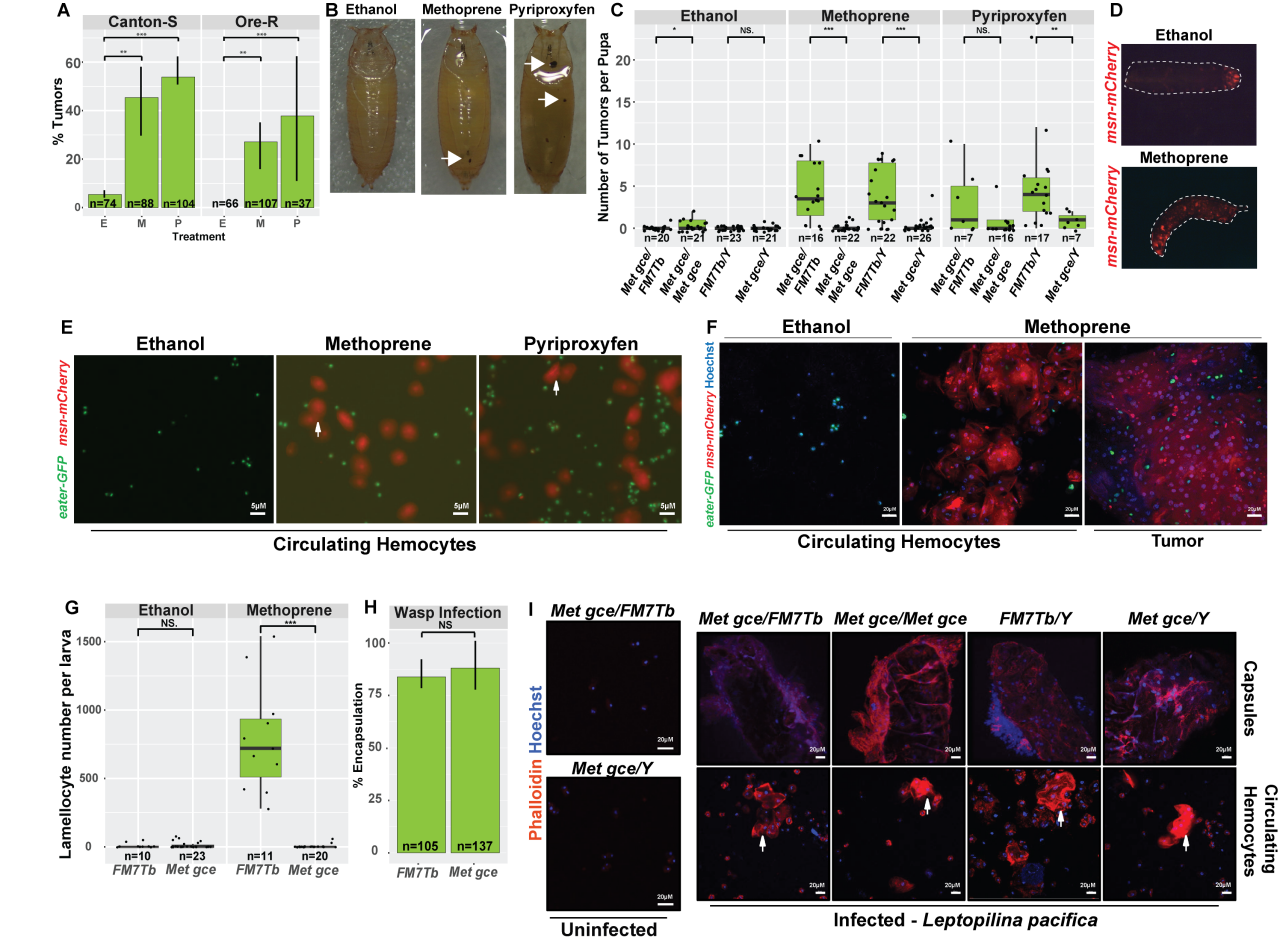
**(A)** Two different JHMs, methoprene (M) and pyriproxyfen (P), induce melanotic tumors in Canton-S and Oregon-R (Ore-R) wildtype larvae at a higher rate than ethanol (E) vehicle alone. Percentage of prepupae with tumors, calculated for each genotype and treatment is shown. Bars represent standard deviation of three biological replicates. Differences between Canton-S and Ore-R tumor induction rates were not statistically significant; Chi-square test. **(B)** Prepupae with melanotic tumors. Arrows point to tumors. **(C)** A mutant strain that lacks sensitivity to JHMs, *Met ^1^ gce ^Mi^* , makes fewer tumors after exposure to methoprene or pyriproxyfen than balancer controls. Points represent the number of tumors per animal (n is the number of animals examined); Wilcox test. **(D)** *In vivo* visualization of a lamellocyte reporter *msn-mCherry* . In an ethanol treated wandering third instar larva, posterior *msn-mCherry* signal is observed, while by 24 hours after methoprene treatment, lamellocytes are found throughout the body. Representative data from more than 6 biological replicates with 10 samples each. **(E, F)** Circulating hemocytes in wandering third instar larva are primarily plasmatocytes (green, *eater-GFP* ) unless treated with methoprene or pyriproxyfen (red, *msn-mCherry* ). Plasmatocytes and lamellocytes can also be identified by cell shape and size. **(F)** Close-up of methoprene induced lamellocytes and a tumor covered in lamellocytes. Nuclei labelled in blue (Hoechst). **(G)** Circulating hemocytes from *Met ^27^ gce ^2.5k^* mutants do not include lamellocytes after methoprene treatment. Each point represents the number of lamellocytes scored for one larva. n is the number of larvae scored; Wilcox test. **(H, I)** Met and gce are not required to encapsulate parasitoid wasp, *Leptopilina pacifica,* eggs. **(H)** Homozygous or hemizygous mutant *Met ^1^ gce ^Mi^* larvae encapsulate wasp eggs at the same rate as controls. Bars represent standard deviation. n is the number of infected larvae examined, from 7 independent infections. (t-test). **(I)** Encapsulated wasp eggs (top) or circulating hemocytes (bottom) from control and mutant wandering third instar larva. Because lamellocytes have high levels of F-actin, strong rhodamine-Phalloidin signal indicates their presence in the melanized capsules. Arrows point to lamellocytes. Red: Phalloidin, Blue: Hoechst. For panels **(A, C, G, H)** p-values: * p<0.05, **p<0.01, ***p<0.001, NS = not significant.

## Description

Juvenile hormone (JH) is an important coordinator of development in insects, moderating the effects of another major hormone, ecdysone, at each molt (Riddiford, 2020). Due to JH’s many roles in development, behavior and reproduction in insects, molecules functionally similar to JH, the juvenile hormone mimics (JHM) methoprene and pyriproxyfen, are widely used insecticides for controlling disease vectors and agricultural pests (Lawler, 2017; Martin et al., 2024). A suite of developmental defects occurs in *Drosophila melanogaster* after JH or JHM exposure (Ashburner, 1970; Riddiford & Ashburner, 1991). These defects include failure to eclose from the pupal case, morphogenesis defects of adult tissues, degeneration of the photoreceptors and production of melanotic tumors (Postlethwait, 1974; Restifo & Wilson, 1998; Wilson & Fabian, 1986). Induction of these tumors can be reduced in *Met ^1^ and Met ^2^* mutants lacking the juvenile hormone receptor, Methoprene-tolerant (Met) (Wilson & Fabian, 1986). Similar looking melanotic tumors have been shown to form in various *Drosophila* mutants, most well-known being *hopscotch ^Tumorous-lethal^* ( *hop ^Tum-l^* ), with constitutively active JAK-STAT immune signaling (Harrison et al., 1995). The *hop ^Tum-l^* tumors are caused due to overproliferation of the hematopoietic tissue and ectopic lamellocyte differentiation (Luo et al., 1995; Panettieri et al., 2019).

The cellular immune system in *D. melanogaster* consists of three major hemocyte cell types. The majority (95%) of the cells are plasmatocytes (10-20 µm) that function as macrophages (Gold & Brückner, 2015). Platelet-like and oxygen carrying crystal cells make up the rest of the hemocytes (Shin et al., 2024). Under assault by parasitoid wasps, a third type of hemocyte, called lamellocytes, is induced. In a classic cellular immune response, these large, flat hemocytes (40-50 µm in size), surround parasitoid wasp eggs that are laid inside the larval hemocoel and encapsulate to kill the parasite, allowing the host to survive (Rizki & Rizki, 1979; Sorrentino et al., 2002).

Ecdysone and JH have known roles in *Drosophila* immunity (Flatt et al., 2008; Keith, 2023; Nunes, Koyama, et al., 2021; Nunes, Sucena, et al., 2021; Regan et al., 2013; Sorrentino et al., 2002; Tian et al., 2010; Zhang & Palli, 2009). Even though the JHM melanotic tumor response has been known for over 40 years, the cell types making up these tumors and the mechanisms underlying their appearance have not been investigated. Based on their apparent similarities in size and locations to blood tumors in *hop ^Tum-l^* mutants, we hypothesized that JHM exposure might affect the fly’s hematopoietic system, increase the abundance of the lamellocytes (even in the absence of parasitoid infection), and thus trigger tumorogenesis. We investigated if lamellocytes are induced by exposure to methoprene or pyriproxyfen and if the induced tumors were composed of lamellocytes.

We tested if two JHMs, methoprene and pyriproxyfen, induce tumors in wildtype *Drosophila* strains, Canton-S and Oregon-R, and found that this phenomenon is induced by both JHMs in a similar manner ( Figure 1A ). A range of small, medium and large tumors arise in both fly strains ( Figure 1B ). *Met* mutants are resistant to both methoprene (Wilson and Fabian, 1986) and pyriproxfen (Riddiford & Ashburner, 1991). Since the original report of methoprene induced melanotic tumors, a second copy of the JH receptor gene was found in the *Drosophila melanogaster* genome, *germ cell expressed* ( *gce* ) (Abdou et al., 2011). To examine their roles in JHM-induced tumorigenesis, we treated a mutant *Met ^-^ gce ^-^* line that lacks both juvenile hormone receptors with JHMs. *Met ^-^ gce ^-^* escapers have reduced fecundity and germ cell migration defects (Barton et al., 2024). *Met ^-^ gce ^-^* larvae produced almost no tumors ( Figure 1C ).

To examine if lamellocytes are a major constituent of the tumors, we utilized an *in vivo* lamellocyte marker, *msn-mCherry* (Tokusumi, Shoue, et al., 2009) to visualize these cells. Under non-immune challenged conditions, third instar larvae do not produce lamellocytes and only the background *msn-mCherry* signal is observed. However, following methoprene treatment, lamellocytes are seen throughout the larvae ( Figure 1D ). Next, we examined circulating hemocytes from larvae with plasmatocytes labelled with *eater* -GFP (Kroeger Jr. et al., 2012) and lamellocytes labelled with *msn-mCherry* . Smears of ethanol treated control larvae contain plasmatocytes, while those of methoprene and pyriproxyfen treated larvae contain both plasmatocytes and lamellocytes ( Figure 1E, F). JHM treated tumors are composed of many lamellocytes ( Figure 1D, F). Thus lamellocytes are not found in ethanol treated controls but are found in large amounts in both methoprene and pyriproxyfen treated animals. After methoprene treatment, *Met ^-^ gce ^-^* larvae did not form lamellocytes, either ( Figure 1G ). These results suggest that the JH receptors mediate the sensitivity to JHM required to induce melanotic tumors made of aggregated lamellocytes.

Since lamellocytes did not form in the *Met ^-^ gce ^-^* mutants under ectopic JHM treatment, we tested if wild type Met and Gce functions are required for lamellocyte differentiation induced upon exposure to the parasitoid wasp *Leptopilina pacifica* (Indonesia, *Lp-Indo* ) (Novković et al., 2011). *Met ^-^ gce ^-^* stocks are maintained with an X-chromosome balancer due to reduced viability and fecundity. The marker Tubby (Tb) was used to distinguish between mutant and control larvae. The encapsulation capacity of internal control FM7a-Tb balancer larvae, shows that 85 ± 6.9% of larvae produced one or more melanized capsule and 89 ± 11.6 of *Met ^-^ gce ^-^* larvae mount a similar response ( Figure 1H ). *Lp-Indo* eggs dissected from *Met ^-^ gce ^-^* larvae are encapsulated by lamellocytes, similar to eggs dissected from the balancer control larvae ( Figure 1I, top panels). The rhodamine-Phalloidin signal in these samples varies due to different capsule morphologies and differing levels of melanization. Lamellocyte morphology is observed more clearly in circulating hemocyte smear preparations ( Figure 1I, bottom panels, arrows). *Met ^-^ gce ^-^* mutants were also capable of encapsulating *Leptopilina boulardi* eggs, 11 ± 9.6% of infected larvae examined compared to 22 ± 27.2% of balancer control; Chi-square test p=0.22.

Our results clearly demonstrate that exposure to either methoprene or pyriproxyfen induces a cellular immune response in *Drosophila* larvae. This response is reproducible and robust. While Met and Gce are required for the ectopic cellular immune responses induced by JHM treatments, the receptors appear to be dispensable for the wasp-induced lamellocyte differentiation response. The simplest interpretation of these observations is that, like *hop ^Tum-I^* , (Bailetti et al., 2019; Panettieri et al., 2019) the JHMs expand the hematopoietic pool that triggers the differentiation of hemocytes. We hypothesize that the JHM sensitivity that induces lamellocytes may be required in a non-hematopoietic tissue such as the fat body or muscles that would then signal to the lymph gland, prohemocytes and hemocytes. Future work will shed more light on the mechanism of action of JHMs on hematopoietic development, as these insecticides become more important in pest control in the warming climate. A role for JH signaling in hematopoietic development cannot be ruled out and needs to be investigated more thoroughly.

## Methods

Fly stocks and rearing

Flies were reared at 25°C on Archon Scientific molasses food (86% water, 0.57% agar, 6.3% cornmeal, 1.5% yeast, 4.7% molasses, 0.4% propionic acid, 0.15% methylparaben, 0.5% ethanol). Each 28.5 mm diameter polystyrene vial contained 12 mL of food (Catalog #B20301).

Oregon-R (Ore-R) BDSC 25211 and Canton-S BDSC 64349 were obtained from the Bloomington Drosophila Stock Center. *Met ^1^ gce ^Mi02742^* /FM7a-Tb (Barton et al., 2024) was a gift from L. Barton. *Met ^27^ gce ^2.5^ /FM7a-Tb* (Abdou et al., 2011); was a gift from L. Riddiford. The *msn-mCherry* (Tokusumi, Sorrentino, et al., 2009), *MSNF9mo-mCherry eater-GFP* (Schmid et al., 2016; Sorrentino et al., 2007) stocks were obtained from R. Schulz and D. Hultmark, respectively. We used FlyBase (release FB2025_03) to find information on mutant phenotypes and *Drosophila* stocks (Öztürk-Çolak et al., 2024).

JHM treatments and tumor scoring

*Drosophila* adults were allowed to lay eggs for a maximum of 48 and then removed. 25 µL of ethanol (control), methoprene (1 µg/µL in ethanol, Chem Service Inc N-12400) or pyriproxyfen (0.2 µg/µL in ethanol, Fisher Scientific NC0050502) was evenly distributed over the vial surface when the larvae reached the late second or early third instar larvae. These doses are similar to those used in (Baumann et al., 2017; Restifo & Wilson, 1998). These doses of methoprene and pyriproxyfen allowed for continued development until pharate adult stage but caused a 100% failure of Canton-S and Ore-R to eclose as adults.

Tumors were scored at wandering third instar and prepupal stages as in (Wilson & Fabian, 1986) using Leica EZ4 stereomicroscope and photographed using LAS EZ software. *In vivo* induction of lamellocytes was observed with the *msn-mCherry* marker. Wandering third instars were placed on a glass dish, put at -20°C for 20 seconds to immobilize them and imaged using a Zeiss Discovery.V8 stereomicroscope.

Hemocytes and imaging

Hemocyte smears, tumors, and wasp capsules from dissected wandering third instar larvae were prepared as follows. Animals were washed in water, 70% ethanol, and phosphate-buffered saline, pH 7.6 (PBS). They were placed on a microscope slide and hemolymph contents were bled, air dried (30 min), and fixed in 4% paraformaldehyde. After washing off the fixative with PBS, hemocytes were stained with Hoechst 33258 (Invitrogen, 1:500) and Rhodamine or Alexa Fluor 488-tagged Phalloidin (Invitrogen). Samples were mounted in VectaShield (Vector Laboratories). Hemocytes were identified by their high F-actin staining signal and distinctive shape. We also utilized *Drosophila* strains with *in vivo* labelled plasmatocytes (visualized with the *eater-GFP* reporter), and lamellocytes (with *msn-mCherry* reporter). These hemocyte smears were counterstained with Hoechst 33258. Samples were imaged using a Zeiss LSM 800 confocal microscope. Images were then imported into FIJI (Schindelin et al., 2012) and converted into a jpg file.

Lamellocyte counting

Hemocytes from *Met ^27^ gce ^2.5^* / *Y* and *FM7c-GFP/Y* wandering third instar larva from the same treated vials were collected as described above. Hemocytes from a single larva were collected in 100 µL of PBS and 10 µL were immediately transferred to hemocytometers (Fisher Scientific NC0435502). Lamellocytes were identified using brightfield microscopy based on morphology and size. Total count was estimated by multiplying the hemocytometer count by 10.

Wasps and encapsulation assays

*Leptopilina pacifica* (Indonesia: *Lp-Indo* from T. Schlenke), (Lue et al., 2021; Novković et al., 2011) wasps were reared on *D. virilis* on corn meal Archon food. *Met ^1^ gce ^Mi^ /FM7a-Tb* flies were allowed to lay eggs for 48 hours. Flies were removed and larvae incubated at 25°C to develop for 48 hours. Late second and early third instar larvae were exposed to *Lp-Indo* wasps for 24 hours. After removing the wasps, the encapsulation response was scored 24 hours later. For encapsulation scoring, larvae were removed from the food, cleaned with 70% ethanol, distilled water and PBS in 9-well plates. Using a stereomicroscope, larvae were dissected individually with forceps by gently tearing the cuticle. Larval contents were examined for the presence of wasp eggs, wasp larvae, melanized aggregates, or melanized capsules. Percent encapsulation was computed as number of infected hosts with melanized capsules and aggregates relative to the number of infected hosts scored.

Data visualization and statistics

Statistical analyses were run in R version 4.4.1 (R Core Team, 2021). Graphs were made with ggplot2 (Wickham, 2016).
